# Polymorphisms of toll-like receptors 2 and 9 and severity and prognosis of bacterial meningitis in Chinese children

**DOI:** 10.1038/srep42796

**Published:** 2017-02-16

**Authors:** Pingping Zhang, Nan Zhang, Linlin Liu, Kai Zheng, Liang Zhu, Junping Zhu, Lina Cao, Yiyuan Jiang, Gang Liu, Qiushui He

**Affiliations:** 1 Department of Infectious Diseases, Beijing Children’s Hospital, Capital Medical University, Beijing 100045, China; 2 Department of Medical Microbiology and Research Centre of Microbiome, Capital Medical University, Beijing 100069, China; 3 Department of Medical Microbiology and Immunology, University of Turku, Turku 20520, Finland

## Abstract

Toll-like receptors (TLRs) play a crucial role in innate immunity, protecting the host from bacterial pathogens. We investigated whether bacterial meningitis (BM) in children was associated with gene polymorphisms in TLR2 (rs3804099), TLR3 (rs3775291 and rs3775290) and TLR9 (rs352139 and rs352140). Blood samples were taken from 218 child patients with confirmed BM and 330 healthy adult controls (HC) and polymorphisms of these genes were analyzed by PCR-based sequencing. For TLR2 rs3804099, frequencies of the minor allele C were markedly higher in patients with severe BM (defined as CSF glucose concentration ≤ 1.5 mmol/L and seizures) than those without (43.5% and 40.1% vs. 30.1% and 29.1%, p = 0.008 and p = 0.016, respectively). For TLR9 rs352139, patients who carried genotype AA and minor allele A developed seizures less often than those without (OR = 0.289, p = 0.003 and OR = 0.568, p = 0.004, respectively). However, for TLR9 rs352140, patients who carried genotype TT and minor allele T developed seizures more often than those without (OR = 3.385, p = 0.004 and OR = 1.767, p = 0.004, respectively). Our finding suggested that genetic variations in TLR2 and TLR9 are associated with severity and prognosis of bacterial meningitis in Chinese children. However, the results should be interpreted with caution since the number of subjects included was limited.

Bacterial infection of the central nervous system (CNS) is an important public health problem worldwide in young children. Despite immunization programs and improvement of antimicrobial treatment and the use of adjunctive therapy such as corticosteroids, CNS infections especially bacterial meningitis (BM) remain a significant cause of pediatric illness and death in low and middle income countries^1^,^2^,^3^. Reported mortality rates are high being from 4% to 10%, and about 20% of BM survivors develop neurological sequelae including learning and behavioral disorders, deafness and motor deficits^4^,^5^. To reduce the neurological complications, it is important to make early diagnosis and treatment and to identify children at high risk of BM.

The pathogenesis of BM involves a complex interplay between bacteria and the host immune response. In most cases, it starts with invasion of the colonized bacteria in the bloodstream^6^,^7^. The invaders cross the blood–brain barrier and then cause infections in the CNS. Another route for bacteria to reach CNS may be through direct contamination of the cerebrospinal fluid (CSF) from indwelling devices, skull fractures, or infections of the nasopharynx or the nasal sinuses that have formed a tract with the subarachnoid space^8^. Once CNS is infected, host defense starts with a complex cascade of bacterial recognition by Toll-like receptors (TLRs) in immune cells of the brain (astrocytes and microglia) and leads to the production of various inflammatory mediators such as tumor necrosis factor-α, interleukins, interferons and nitric oxide, which are toxic to the replication of bacteria^7^,^9^. On the other hand, large amount of cytokines in CNS causes inflammation of the wall of blood vessels, which leads to increased blood-brain barrier permeability and decreased cerebral blood flow perfusion, consequently brain cells deprived of oxygen and undergo apoptosis (inflammation associated tissue damage)^10^. In view of this, early recognition of invading bacteria by TLRs and the shaping of appropriate immune responses are essential for control of BM development.

Growing data indicate that the ability of certain individuals to respond properly to the pathogens may be impaired due to genetic variation of TLR genes^11^. It has been shown that TLR2 plays a significant role in a murine model of experimental pneumococcal meningitis and the deficient TLR2 leads to reduced bacterial clearance from the CNS^12^,^13^. In human, homozygous variant of TLR2 rs3804099 (C597T) was found to be associated with susceptibility to tuberculous meningitis in Vietnamese^14^. Recently Zhang *et al*. have reported that healthy Chinese subjects who carried genotypes of TLR2 rs3804099 CT/TT had a superior innate immune response to *Legionella pneumophila*^15^. In CNS of both human and murine, TLR3 is mostly expressed in astrocytes, that is considered to be closely linked to anti-viral signaling, as it results in the production of type I interferon (IFN)^16^,^17^. TLR9 recognizes DNA unmethylated CpG motifs from bacteria and viruses^18^. Mice deficient in TLR9 have reduced survival with the intranasal challenge of *Streptococcus pneumoniae* compared to wildtype mice^19^. In human SNPs in TLR9 were associated with meningococcemia and meningococcal meningitis in Dutch children^20^,^21^. Altogether, data from these studies suggest that bacterial infection leads to a TLR-dependent innate response, and functional variations in TLRs can influence susceptibility and outcome of various bacterial infections.

Although a vast number of TLR SNPs have been identified, there is no reported study to investigate the possible associations between the TLR SNPs and susceptibility, severity or prognosis of BM in Chinese children. The present study was aimed to investigate whether there is an association between gene polymorphisms in TLR2, TLR3 or TLR9 and BM and to identify patients at risk for severe disease and sequelae. Candidate SNPs were chosen based on published data of experimental studies in mice and of known genetic variation of TLRs and their possible effects on susceptibility to meningitis in human^14^,^21^,^22^,^23^.

## Results

### Association between BM susceptibility and gene polymorphisms of TLRs

For susceptibility analysis of genotyping results, BM and HC were compared to each other. The genotypes and allele frequencies of 5 TLR SNPs in cases and controls did not differ significantly, except TLR3 rs3775291 TT genotype (Table 1). For TLR3 rs3775291, lower frequency of TT genotype in BM patients (3.7%) was found when compared to that of HC (9.1%) (OR = 0.366, 95%CI: 0.162–0.826, P = 0.013).

No significant differences were noticed between BM patients and HC when combined analyses of different genotypes of TLR9 rs352139 and rs352140 were performed.

To confirm accuracy of genotypes for each SNP, 20 of DNA samples were randomly selected and tested in duplicate. The results obtained were identical. The measured 5 SNPs were tested to be in Hardy-Weinberg equilibrium (all p values > 0.05).

### Demographical and clinical details of study subjects

A total of 218 pediatric patients (median age: 4 months; range: 3 months to 14 years) with clinically confirmed BM were included. Of them, 106 (48.6%) were etiologically confirmed (Table 2). Bacterial pathogens were identified based on a positive culture and/or antigen detection obtained in the CSF and/or blood. Of 106 positive samples, 89 were positive from CSF and 30 were from blood. *S. pneumoniae* (n = 37, 34.9%) and Group B Streptococcus (n = 23, 21.7%) were the most frequently detected pathogens. Because different pathogens may have different effect on severity and prognosis of BM, we analyzed the pathogen distribution in patients with different genotypes of TLRs studied and did not find any association (data not shown).

All 218 BM patients had fever at admission. No difference in genders was found between BM patients and HC. Poor prognosis was documented in 15.4% of BM cases. As expected, laboratory tests of CSF consisted of pleocytosis (median: 215.00 × 10^6^/L; range: [40.00–1050.00] × 10^6^/L) with polymorphonuclear cells predominated (69.0%), decreased glucose concentration (median: 2.20 mmol/L; range 1.15–2.99 mmol/L), and elevated levels of CSF protein (median: 1165.15 mg/L: range 609.75–2169.75 mg/L) were found in BM patients. The detailed information was summarized in Table 2.

### Association between clinical characteristics and gene polymorphisms of TLRs

To study whether gene polymorphisms of the TLRs studied could influence severity and prognosis of BM, we analyzed data based on nine clinical features that might be considered as predictors of BM severity and prognosis^24^. These clinical features included consciousness, seizures, subdural effusion, bacteremia, hearing loss, long-term prognosis, white cell in CSF, glucose in CSF and protein in CSF.

For TLR2 rs3804099, genotype CC was significantly higher in BM patients with an initial CSF glucose concentration of less than 1.5 mmol/L compared to those with greater than 1.5 mmol/L (25.8% vs. 7.3%, P = 0.001) (Table 3). Minor allele C were significantly higher in BM patients with seizures compared to those without (40.1% vs. 29.1%; P = 0.016). For TLR9 rs352139, genotype AA and minor allele A were significantly higher in BM patients with bacteremia compared to those without (41.5% and 66.9% vs. 30.3% and 55.3%; P = 0.016 and P = 0.024) (Table 4). Genotype AA and minor allele A were significantly lower in BM patients with seizures compared to those without (25.3% and 51.1% vs. 40.2% and 64.8%; P = 0.003 and P = 0.004). For TLR9 rs352140, genotype TT and minor allele T were significantly lower in BM patients with bacteremia compared to those without (7.7% and 33.1% vs. 19.7% and 44.1%; P = 0.020 and P = 0.033) (Table 5). Genotype TT and minor allele T were significantly higher in BM patients with seizures compared to those without (22.9% and 48.4% vs. 10.7% and 34.7%; P = 0.004 and P = 0.004).

For TLR3 rs3775291, the minor allele T carriers (CT+TT) were associated with a decreased incidence of subdural effusion (OR = 0.578, 95%CI: 0.337–0.992, P = 0.046) and seizures (OR = 0.500, 95%CI: 0.279–0. 894, P = 0.030) during the course of BM (Data not shown). No significant differences were found in other clinical characteristics between patients with different genotypes (Data not shown).

The SNPs found to be statistically significant in univariate analysis were further analyzed by logistic regression. Most of significant differences observed by univariate analysis remained statistically significant. After logistic regression analyses were applied, significant differences observed between variant genotypes (CT+TT) of TLR3 rs3775291 and subdural effusion, between the CC genotype of TLR2 rs3804099 and poor prognosis, between the minor allele C carriers (TC+CC) of TLR2 rs3804099 and seizures became not significant.

### Treatment of patients and gene polymorphisms of TLRs

In clinical practice in China, vancomycin or meropenem are usually given at admission when patients are clinically diagnosed as having BM, followed by the third or fourth generation cephalosporin in combination of vancomycin or not based on antimicrobial sensitivity result once bacterial pathogen(s) are confirmed. For laboratory-confirmed patients, sensitive antibiotics were selected with the guidance of drug sensitivity test. If the outcome of patients is not improved, Gamma globulin might be used as adjuvant treatment. Steroid might be chosen for BM critical cases at very early stage. To study possible effect of treatment on disease course and outcome in patients with and without SNPs, we compared **v**arious forms of treatment mentioned above with different genotypes of BM patients from whom significant difference was found in certain clinical features (Table 3 to Table 5). No significant differences were observed.

## Discussion

To our knowledge, this is the first study carried out in China to show the relationships between TLRs and susceptibility, severity and prognosis of bacterial meningitis in pediatric patients. An association between TLR2 polymorphism and BM susceptibility was not observed. Instead we found patients with genotype CC of TLR2 rs3804099 had at least four fold increased risk to have lower concentration of glucose in CSF, indicating disease severity and poor prognosis. Multicenter studies have indicated that hypoglycorrhachia is an important predictor of severity during the CNS infection as well as important mortality indicator in BM^25^,^26^. The insufficient glucose in the CSF can greatly affect neuron energy supply and cause damage to the brain. Furthermore, low levels of glucose in the CNS has been associated with increased inflammation and cytokine levels in the CSF which may consequently results in the pathogenesis of meningitis-associated brain injury, including neurological sequelae and seizures generation^27^,^28^. Seizures then will cause brain cells to consume more energy, in the form of glucose, and eventually lead to further reduction of glucose in the CSF^29^.

In this present study, TT genotype of TLR3 rs3775291 seemed to be associated with decreased susceptibility to BM as well as the variant genotypes (CT+TT) with reduced incidence of seizures during the course of BM. Since detection of viruses was not performed in the study subjects and a proportion of patients may have suffered from co-viral infection, the association observed between TLR3 polymorphism and BM patients should be interpreted with caution. However it should be kept in mind that all these BM patients were clinically improved after treatment by antibiotics. Further investigation with the purpose to identify causative agents of bacteria and viruses in a large population is needed.

In respect to polymorphisms of TLR9, no differences in frequencies of genotypes or alleles were observed between BM patients and HC. We even could not find any significant differences when combined analysis of the two SNPs of TLR9 was performed. Our results indicated that SNPs of TLR9 might not be associated with susceptibility to BM in Chinese children. This finding seems to be different from those of other studies conducted in Indonesians and Mexicans with tuberculosis, as well as in Dutch children with meningitis caused by *Neisseria meningitides*^21^,^30^. It should be kept in mind that the sample size, ethnics and bacterial characteristics of our study population are different from the two studies mentioned above. Interestingly, we found that patients who carried genotypes GA and AA of TLR9 rs352139 developed seizures less often than those without, whereas patients who carried genotype TT of TLR9 rs352140 developed seizures more often than those without. A recent study has indicated that TLR9 deficiency may exacerbate seizure-induced cognitive decline and recurrent seizure severity in mice^31^. It is known that seizures often occur as a response to cerebral injury during CNS infection, and brain inflammation is a crucial predisposing factor of seizure susceptibility^32^,^33^,^34^. Recent studies in animal models suggest that inflammation induced seizures would in turn up-regulate brain-borne inflammatory mediators (IL-1β and CCL2), aggravating brain inflammation and promoting blood-brain barrier (BBB) damage^35^,^36^. BBB leakage was known to be associated with altered microenvironment of neurons, which contributes to enhanced excitability and reduced firing threshold for seizures^37^. There is even evidence showing the possibility of aggravated neuronal injury after only a single seizure^38^. The vicious circle of brain inflammation, BBB dysfunction and seizures worsened cerebral inflammation, finally resulted in inflammation associated brain damage in the CNS, thereby presented with more severe manifestations (seizures) or even neurological sequelae. However, seizures induce brain inflammation and BBB damage independently on extracerebral factors like blood-borne inflammatory molecules, and brain-borne inflammation is unique and sufficient to maintain seizure activity^36^. This indicates that the vicious circle of excessive inflammation response was only presented inside the CNS but not in the blood. So children with TLR9 SNPs may have a lower chance to develop bacteremia because of the adequate but balanced bactericidal effects and higher chance of developing seizures due to the excessive inflammation associated brain damage in the CNS. It is known that *S. pneumoniae* is one major pathogen causing bacterial meningitis^11^. Recent study have shown that bacterial load of S. pneumonia in the blood was associated with brain damage and outcome of BM in experimental animals^39^,^40^. Our study was focused on host genetics of TLRs and severity and outcome of children with clinical confirmed BM. Unfortunately data on bacterial load in blood and CSF were not studied. Further study is needed to confirm whether the bacterial load in the blood is linked to severity and outcome of BM patients.

In this study, all treatments for patients were conducted before genotyping of TLRs was performed. Thus doctors did not know genotypes patients carried when treatment was given, and the type and length of treatment given only depended on the condition of patients, suggesting that there was no treatment bias for BM children with different genotypes. When various forms of treatment in patients with different genotypes of TLRs were analyzed, we did not find any associations.

There were certain limitations in this study. First, the number of patients included was limited. Second, the healthy adults used as controls in this study are not optimal. As stated, the purpose to include the adult controls was not meant to compare these clinical features and outcome of BM between child patients and adult controls. The aim was to investigate if there is difference in genotype distributions of these SNPs of TLRs studied between the two groups of subjects, as the genotype distributions of the SNPs are not dependent on the age of subjects. Third, only half of patients were etiologically confirmed. However, as mentioned, clinical diagnosis of patients were made strictly based on the WHO guidelines by an experienced physician^41^, and laboratory results including peripheral blood neutrophil predominated, polymorphonuclear pleocytosis, low CSF glucose concentration and high CSF protein levels at admission also supported the diagnosis made for BM. A recent study carried out in the Beijing Children’s Hospital among 507 children with BM during the period of 2010–2014 found that only 43.4% of patients were etiologically confirmed and 63.9% had used antibiotics before they were hospitalized^42^. The positivity rates of pathogens detected in BM patients were almost the same as that found in our study. Moreover, distributions of common pathogens found in the two studies were also similar.

Our finding supports a role of genetic variation of TLR2 and TLR9 in severity and prognosis of Chinese Han children to bacterial meningitis. More association and functional studies on polymorphisms of TLR are needed to reveal the exact mechanism causing the differences in clinical course of BM and to obtain genetic traits which can be used for patient profiling and management of CNS infectious patients.

## Methods

### Study subjects

Padiatric patients (age range, 1 month to 18 years) with clinically confirmed BM were enrolled from an on-going study of CNS infections, which was started in January 2014 at Department of Infectious Diseases, Beijing Children’s Hospital (a 970-bed tertiary health care hospital), Beijing, China. The control group consisted of 330 healthy adults who attended annual medical examination in June 2015. The median age was 38 years (range from 21 to 60 years) and gender ratio of these adults was 1.04 (male vs. female: 168/162). Written informed consent was taken prior to the study enrollment from all study subjects, or their guardians in the case of young children who were unable to write or consider consent independently. Clinical diagnosis of BM patients was performed by trained medical personnel at the department according to the guidelines of the World Health Organization (WHO)^41^. All the subjects included in this study were found to have good clinical response (e.g, fever curve, resolution of symptoms and signs) after 24 to 36 hours of empirical antibiotics therapy. All study subjects were Chinese from different families and thus considered as unrelated individuals.

The study was approved by the Ethics Committee of the Capital Medical University and Beijing Children’s Hospital of Capital Medical University, Beijing, China. The methods were carried out in accordance with the relevant guidelines, including any relevant details.

### Clinical data collection

Clinical characteristics of the study subjects were summarized in Table 2. Ethnicity was based on electronic medical records (EMR) and categorized as Chinese Han descendents. Clinical information regarding fever, consciousness, seizures, subdural effusion and hearing loss were provided by one medical doctor (GL) at the department of Infectious Diseases. Other clinical information on C-reactive protein (CRP), cerebrospinal fluid (CSF) white cell counts, blood white cell (WBC) numbers, duration of hospitalization and treatment were all collected from the EMR. A long-term follow-up study was conducted by one study personnel (PZ) through phone call or follow up visit by Glasgow Outcome Scale–Extended Pediatric Revision (GOS-E Peds), which provides the gold standard for measuring meningitis associated brain damage outcome^43^. Patients would be considered as having good prognosis if GOS-E Peds ≤ 2, otherwise poor prognosis.

### Samples collection and bacterial culture

At admission, both CSF and blood samples were taken aseptically from each study subject before antibiotic therapy. In addition to the routine biochemical analysis, bacterial culture, Gram staining, acid fast staining and *S. pneumoniae* antigen test were performed immediately at the Microbiology laboratory to identify etiologic agent. All the bacterial classification and identification were based on the standard laboratory identification method^44^. Antibiotic susceptibility test was performed based on the minimum inhibitory concentration (MIC) values of cultured bacterial isolates. Since all the subjects of this study were strictly included according to the WHO diagnostic standards for BM and their conditions were clinically improved after the treatment with antibiotics, detection of viruses was not performed. In this present study, multiple bacterial infections were not detected.

### DNA extraction

Genomic DNA was extracted from 200 μL of blood by using a DNA purification kit (QIAamp DNA Blood Mini Kit; Qiagen, Valencia, CA) according to manufacturer’s instructions. The extracted DNA was stored at −20 °C until used.

### SNPs analysis

The SNP detection of TLR2 (T597C) rs3804099, TLR3 (C1235T) rs3775291, TLR3 (C1377T) rs3775290, TLR9 (A6808G) rs352139 and TLR9 (C8483T) rs352140 were performed by PCR-based sequencing. The primers used for PCR and sequencing were earlier described^14^,^21^,^22^,^23^. The total reaction volume of 50 μL contained 5 μL of 10× buffer, 1 μL of each primer, 2 μL of genomic DNA, 0.5 μL of dNTP mixture, 0.5 μL of Taq DNA Polymerase and 40 μL of deionized water. Amplification conditions consisted of initial denaturation at 95 °C for 2 min followed by 42 cycles of 95 °C for 30 s, 55 °C (slightly different with different primer sets) for 30 s, and 72 °C for 2 min, with a final extension for 5 min at 72 °C. SNPs of each TLR were detected by PCR-based sequencing. Positive and negative controls were used in each PCR run. To confirm genotyping result obtained for each SNP, 20 of samples were randomly selected and tested in duplicate.

### Statistical analysis

Allelic and genotypic frequencies of each SNP studied were calculated. All of the SNPs analyzed were found to be in HWE (P > 0.05). Univariate analysis was performed for continuous variables with Student’s t-test and categorical variables with χ^2^ test. Fisher’s exact test was used when the number of samples in a group was less than five. The Odds Ratio (OR) and 95% confidence interval (CI) were calculated using unconditional binary logistic regression. Genotypes found to be statistically significant by univariate analysis were further analyzed by logistic regression. A two-tailed P value less than 0.05 was considered significant. All the statistical analyses were conducted using the SPSS software, version 17.0. The genotype distribution was analyzed for deviations from the Hardy–Weinberg equilibrium (HWE) using the goodness-of-fit χ2 test, performed by using a professional web-based program (http://ihg.gsf.de/cgi-bin/hw/hwa1.pl).

## Additional Information

**How to cite this article**: Zhang, P. *et al*. Polymorphisms of toll-like receptors 2 and 9 and severity and prognosis of bacterial meningitis in Chinese children. *Sci. Rep.* **7**, 42796; doi: 10.1038/srep42796 (2017).

**Publisher's note:** Springer Nature remains neutral with regard to jurisdictional claims in published maps and institutional affiliations.

## Figures and Tables

**Table 1:** Genotype and allele frequencies of TLR2 (T597C) rs3804099, TLR3 (C1235T) rs3775291, TLR3 (C1377T) rs3775290, TLR9 (A6808G) rs352139 and TLR9 (C8483T) rs352140 in BM patients and population-based controls*.

Genotypes and allele frequencies	Cases, No. (%)	Controls, No. (%)
TLR2 rs3804099 TT	97/218 (44.5)	148/330 (44.8)
TLR2 rs3804099 TC	94/218 (43.1)	148/330 (44.8)
TLR2 rs3804099 CC	27/218 (12.4)	34/330 (10.4)
TLR2 rs3804099 major allele T	288/436 (66.1)	444/660 (67.3)
TLR2 rs3804099 minor allele C	148/436 (33.9)	216/660 (32.7)
TLR3 rs3775291 CC	116/218 (53.2)	159/330 (48.2)
TLR3 rs3775291 CT	94/218 (43.1)	141/330 (42.7)
TLR3 rs3775291 TT	8/218 (3.7)	30/330 (9.1)
TLR3 rs3775291 major allele C	326/436 (74.8)	459/660 (69.5)
TLR3 rs3775291 minor allele T	110/436 (25.2)	201/660 (30.5)
TLR3 rs3775290 CC	100/218 (45.9)	159/330 (48.2)
TLR3 rs3775290 CT	92/218 (42.2)	141/330 (42.7)
TLR3 rs3775290 TT	26/218 (11.9)	30/330 (9.1)
TLR3 rs3775290 major allele C	292/436 (67.0)	459/660 (69.5)
TLR3 rs3775290 minor allele T	144/436 (33.0)	201/660 (30.5)
TLR9 rs352139 GG	35/217 (16.1)	58/330 (17.6)
TLR9 rs352139 GA	109/217 (50.3)	166/330 (50.3)
TLR9 rs352139 AA	73/217 (33.6)	106/330 (32.1)
TLR9 rs352139 major allele G	179/434 (41.2)	282/660 (42.7)
TLR9 rs352139 minor allele A	255/434 (58.8)	378/660 (57.3)
TLR9 rs352140 CC	75/217 (34.6)	213/597 (35.7)^**^
TLR9 rs352140 CT	107/217 (49.3)	295/597 (49.4)
TLR9 rs352140 TT	35/217 (16.1)	89/597 (14.9)
TLR9 rs352140 major allele C	257/434 (59.2)	721/1194 (60.4)
TLR9 rs352140 minor allele T	177/434 (40.8)	473/1194 (39.6)

*There were no statistically significant differences in genotype and allele frequencies of the SNPs studied between cases and controls, except genotype TT of TLR3 rs3775291 (p = 0.013). **A number of 597 healthy adult controls were tested for TLR9 rs352140.

**Table 2:** Clinical and laboratory information of study subjects.

Characteristics	Patients with BM^※^
Clinical features	
Fever*	218 (100)
Seizures	96 (44.04)
Hospitalization time (days)	20.00 (14.00–31.00)
Level of consciousness at admission	
Normal consciousness	149 (68.66)
Disturbed consciousness	68 (31.34)
Laboratory variables	
CRP (mg/L)	47.35 (8.00–128.73)
Glucose in CSF (mmol/L)	2.20 (1.15–2.99)
Proteins in CSF (mg/L)	1165.15 (609.75–2169.75)
White cell count in CSF (×10^6^/L)$	215.00 (40.00–1050.00)
Polymorphonuclear cells (%)	69.00 (38.00–80.00)
Mononuclear cells or lymphocytes (%)	31.00 (20.00–62.00)
White blood cell count ( × 10^6^/L)$	11.86 (7.93–16.15)
Neutrophils (%)	54.50 (37.15–68.03)
Lymphocytes (%)	33.80 (20.00–49.20)
Pathogen identified	106 (48.6)
Gram positive bacteria	79 (74.5)
*Streptococcus pneumoniae*	37 (34.9)
*Group B Streptococcus*	23 (21.7)
*Staphylococcus aureus*	5 (4.7)
*Listeria monocytogenes*	4 (3.8)
Others^a^	10 (9.4)
Gram negative bacterium	27 (25.5)
*Escherichia coli*	11 (10.4)
*Salmonella*	4 (3.8)
*Haemophilus influenzae type b*	3 (2.8)
*Enterococcus*	3 (2.8)
*Neisseria meningitides*	2 (1.9)
Others^b^	4 (3.8)
Long-term prognosis	
Good prognosis	170 (77.98)
Poor prognosis	31 (14.22)
Lost to follow up	17 (7.80)

^※^Absolute count (%) for categorical variables and median (IQR) for continuous data, unless otherwise stated.

^*^Axillary temperature >38.0 °C.

^$^For patients for whom CSF and blood samples were obtained in the first 7 days of hospitalization.

^a^Including Streptococcus oralis, 1 subject; Streptococcus mitis, 1 subject; Streptococcus dysgalactiae, 1 subject and Gram positive bacteria indentified in CSF smear, 7 subjects.

^b^Including Klebsiella pneumonia, 1 subject; Proteus mirabilis, 2 subjects and Gram negative bacteria identified in CSF smear, 1 subject.

Abbreviations: BM, bacterial meningitis; CRP, c-reactive protein; CSF, cerebrospinal fluid.

**Table 3:** Clinical characteristics in BM patients and genotypes and allele frequencies of TLR2 rs3804099^※^.

Characteristics	Patients with symptoms or parameters defined (%)^&^		OR (95%CI)	P value^#^
**Seizures**	Yes (n = 96)	No (n = 122)		
TT	33 (34.4)	64 (52.5)	1	Ref.
TC	**49** (**51.0**)	**45** (**36.9**)	**2.112** (**1.179–3.784**)	**0.011**
CC	14 (14.6)	13 (10.7)	0.479 (0.202–1.136)	0.091
T	115 (59.9)	173 (70.9)	1	Ref.
C	**77** (**40.1**)	**71** (**29.1**)	**1.631** (**1.094–2.433**)	**0.016**
**Bacteremia**	Yes (n = 65)	No (n = 153)		
TT	27 (41.5)	70 (45.8)	1	Ref.
TC	27 (41.5)	67 (43.8)	0.957 (0.510–1.797)	0.892
CC	11 (16.9)	16 (10.5)	0.561 (0.231–1.362)	0.198
T	81 (62.3)	207 (67.6)	1	Ref.
C	49 (37.7)	99 (32.4)	1.265 (0.824–1.941)	0.281
**Glucose in CSF** (**mmol/L**)*	≤ 1.5 (n = 62)	>1.5 (n = 151)		
TT	24 (38.7)	71 (47.0)	1	Ref.
TC	22 (35.5)	69 (45.7)	1.060 (0.544–2.065)	0.864
CC	**16** (**25.8**)	**11** (**7.3**)	**4.303** (**1.756–10.545**)	**0.001**
T	70 (56.5)	211 (69.9)	1	Ref.
C	**54** (**43.5**)	**91** (**30.1**)	**1.789** (**1.161–2.755**)	**0.008**

^※^There were no statistically significant differences between SNPs of TLR2 rs3804099 in other clinical characteristics including consciousness, subdural effusion, hearing loss, CSF white cell numbers and CSF proteins (all p > 0.05), except long-term prognosis (CC vs. TT+TC, P = 0.031, OR = 3.130, 95%CI:1.213–8.076).

^*^Information was only available in 213 subjects.

Abbreviations: CSF, cerebrospinal fluid; OR, odds ratio; CI, confidence interval.

**Table 4:** Clinical characteristics in BM patients and genotypes and allele frequencies of TLR9 rs352139^※^.

Characteristics	Patients with symptoms or parameters defined (%)^&^		OR (95%CI)	P value^#^
**Seizures**	Yes (n = 95)	No (n = 122)		
GG	22 (23.2)	13 (10.7)	1	Ref.
GA	49 (51.6)	60 (49.2)	2.072 (0.947–4.532)	0.065
AA	**24** (**25.3**)	**49** (**40.2**)	**0.289** (**0.125–0.672**)	**0.003**
G	93 (48.9)	86 (35.2)	1	Ref.
A	**97** (**51.1**)	**158** (**64.8**)	**0.568** (**0.386–0.836**)	**0.004**
**Bacteremia**	Yes (n = 65)	No (n = 152)		
GG	5 (7.7)	30 (19.7)	1	Ref.
GA	33 (50.8)	76 (50.0)	2.604 (0.929–7.299)	0.062
AA	**27** (**41.5**)	**46** (**30.3**)	**3.522** (**1.221–10.158**)	**0.016**
G	43 (33.1)	136 (44.7)	1	Ref.
A	**87** (**66.9**)	**168** (**55.3**)	**1.637** (**1.066–2.519**)	**0.024**
**Glucose in CSF** (**mmol/L**)*	≤ 1.5 (n = 61)	>1.5 (n = 151)		
GG	8 (13.1)	26 (17.2)	1	Ref.
GA	32 (52.5)	75 (49.7)	1.387 (0.567–3.390)	0.472
AA	21 (34.4)	50 (33.1)	1.365 (0.532–3.502)	0.517
G	48 (39.3)	127 (42.1)	1	Ref.
A	74 (60.7)	175 (57.9)	0.894 (0.582–1.373)	0.608

^※^There were no statistically significant differences between SNPs of TLR9 rs352139 in other clinical characteristics including consciousness, subdural effusion, hearing loss, CSF white cell numbers, CSF proteins and long-term prognosis (all p > 0.05).

^*^Information was only available in 212 subjects.

Abbreviations: CSF, cerebrospinal fluid; OR, odds ratio; CI, confidence interval.

**Table 5:** Clinical characteristics in BM patients and genotypes and allele frequencies of TLR9 rs352140^※^.

Characteristics	Patients with symptoms or parameters defined (%)^&^		OR (95%CI)	P value^#^
**Seizures**	Yes (n = 96)	No (n = 121)		
CC	25 (26.0)	50 (41.3)	1	Ref.
CT	49 (51.0)	58 (47.9)	1.690 (0.916–3.117)	0.092
TT	**22** (**22.9**)	**13** (**10.7**)	**3.385** (**1.466–7.817**)	**0.004**
C	99 (51.6)	158 (65.3)	1	Ref.
T	**93** (**48.4**)	**84** (**34.7**)	**1.767** (**1.199–2.603**)	**0.004**
**Bacteremia**	Yes (n = 65)	No (n = 152)		
CC	27 (41.5)	48 (31.6)	1	Ref.
CT	33 (50.8)	74 (48.7)	0.793 (0.424–1.481)	0.466
TT	**5** (**7.7**)	**30** (**19.7**)	**0.296** (**0.103–0.853**)	**0.020**
C	87 (66.9)	170 (55.9)	1	Ref.
T	**43** (**33.1**)	**134** (**44.1**)	**0.627** (**0.408–0.964**)	**0.033**
**Glucose in CSF** (**mmol/L**)*	≤ 1.5 (n = 61)	>1.5 (n = 151)		
CC	22 (36.1)	51 (33.8)	1	Ref.
CT	31 (50.8)	74 (49.0)	0.971 (0.506–1.865)	0.930
TT	8 (13.1)	26 (17.2)	0.713 (0.279–1.820)	0.479
C	75 (61.5)	176 (58.3)	1	Ref.
T	47 (38.5)	126 (41.7)	0.875 (0.569–1.346)	0.544

^※^There were no statistically significant differences between SNPs of TLR9 rs352140 in other clinical characteristics including consciousness, subdural effusion, hearing loss, CSF white cell numbers, CSF proteins and long-term prognosis (all p > 0.05).

^*^Information was only available in 212 subjects.

Abbreviations: CSF, cerebrospinal fluid; OR, odds ratio; CI, confidence interval.
